# Maternal diet influences fecundity in a freshwater turtle undergoing population decline

**DOI:** 10.1093/conphys/coae033

**Published:** 2024-05-27

**Authors:** Kristen Petrov, James U Van Dyke, Arthur Georges, Claudia Keitel, Ricky-John Spencer

**Affiliations:** School of Science, Hawkesbury Institute, Western Sydney University Locked Bag, 1797, Penrith South DC, NSW 2751, Australia; Centre for Freshwater Ecosystems, Department of Environment and Genetics, School of Agriculture, Biomedical and Environment, La Trobe University, Albury-Wodonga Campus, PO Box 821, Wodonga, VIC 3689, Australia; Institute for Applied Ecology, University of Canberra, 11 Kirinari Street,Bruce, ACT 2601, Australia; School of Life and Environmental Sciences, University of Sydney, 380 Werombi Road, Brownlow Hill, NSW 2570, Australia; School of Science, Hawkesbury Institute, Western Sydney University Locked Bag, 1797, Penrith South DC, NSW 2751, Australia

**Keywords:** *Emydura macquarii*, food availability, reproductive output, stable isotopes

## Abstract

Food availability determines the amount of energy animals can acquire and allocate to reproduction and other necessary functions. Female animals that are food limited thus experience reduced energy available for reproduction. When this occurs, females may reduce frequency of reproductive events or the number or size of offspring per reproductive bout. We assessed how maternal diet affects reproductive output in adult female Murray River short-necked turtles, *Emydura macquarii,* from four wetlands in Victoria. We previously found that turtle diets differ in the composition of plants and animals between our study wetlands. In this study, we tested whether differences in turtle diet composition (i.e. plants and animals) at these wetlands were associated with differences in clutch mass, individual egg mass, bulk egg composition and hatching success. We found total clutch mass increased with maternal body size at each site. At sites where filamentous green algae were scarce and *E. macquarii* were carnivorous, females produced smaller clutches relative to body size compared to females from sites where algae were abundant, and turtles were more herbivorous. Individual egg mass, bulk egg composition and hatching success did not differ across wetlands. Isotopic analysis revealed significant positive relationships between the carbon and nitrogen isotopes (δ^13^C, δ^15^N) of the eggs and those of the mothers, indicating that mothers allocated ratios of carbon and nitrogen isotopes to their eggs similar to those present in their tissues. Our study suggests that at sites where females are more carnivorous due to a relative absence of algae, females produce smaller clutches, but other aspects of their reproduction are not significantly impacted. The reduction in clutch size associated with differences in the availability of dietary plants and animals may have long-term consequences for *E. macquarii* and other freshwater turtle species that are experiencing population declines.

## Teaser

Maternal diet affects reproductive output in female freshwater turtles (*Emydura macquarii*). At wetlands where filamentous green algae are scarce and turtles are functionally carnivorous, females produce smaller clutches. Reduced clutch size associated with food availability may have implications for declining freshwater turtle species.

## Introduction

When food is abundant, available, and not limiting, an animal can theoretically allocate sufficient energy to every physiological and behavioural process necessary to maximize fitness (Congdon *et al.,* 1982; Dunham *et al.,* 1989). However, when food is limited, maximizing fitness requires trade-offs in energy allocation among these processes. When unable to gather food sufficient to maintain all of these processes, adult animals may (i) reduce their allocation to growth and sacrifice future (size-dependent) fecundity in order to meet immediate needs, (ii) cease or reduce reproduction until conditions and food availability improves and (iii) reduce the allocation to maintenance and other allocations with a direct bearing on survival such as immune responses. In long-lived species, there are limits to how much of the allocation of available energy directed to processes affecting immediate survival can be reduced (Stoeckmann and Garton, 2001; Beaupre, 2007). A trade-off arises between fitness gain of reproduction in the immediate or short term, and loss of opportunity to capitalize on the fitness benefits of reproduction in future years, should the mother perish (Stoeckmann and Garton, 2001; Beaupre, 2007). The outcome of these trade-offs can explain the evolution of species that fall on the spectrum from semelparity to extreme iteroparity (Bonnet, 2011), where most turtles arguably reside.

Availability of food, moderated by stored resources, determines the amount of energy a female animal can allocate to reproduction at the time of a reproductive event (Congdon, 1989; Bernardo, 1996; Mousseau and Fox, 1998). Most research on the effects of food limitation on animals specifically focuses on the consequences of energy limitation. However, food limitations (i.e. reductions in the amount eaten or changes to the composition of the diet) can also reduce the total nutritional content available to an animal (Ramsay and Houston, 1997). Based on the availability of energy or essential nutrients, females may vary the frequency of reproductive events per year (or over longer time periods), the number of offspring produced and/or the size of offspring (Congdon, 1989; James and Whitford, 1994; Shine and Madsen, 1997; Warner *et al.,* 2007; Van Dyke and Griffith, 2018). Theory predicts that a trade-off also occurs between the number and size of offspring produced, with females either producing many small offspring or few large offspring (Smith and Fretwell, 1974; Rollinson and Hutchings, 2013; Stahlschmidt and Adamo, 2015).

Food type (i.e. plant, animal or both) eaten by females during reproduction or during a prior accumulation period may also affect the availability of nutrients to offspring during development. Carnivory is typically associated with higher consumption of protein and calcium (Clark and Gibbons, 1969; Sánchez-Vázquez *et al.,* 1999), whereas herbivory is associated with higher consumption of fat, carbohydrates, potassium and sodium (King, 1996; Sánchez-Vázquez *et al.,* 1999; Sterner and Elser, 2002). Likewise, food availability may also affect the allocation of essential and non-essential nutrients (especially amino acids and fatty acids) to offspring (Guisande *et al.,* 1999). Under food limitation, essential nutrients (those which cannot be synthesized from other nutrients and must be present in the diet), may be reduced or absent (Wu, 2009). In contrast, non-essential nutrients can be synthesized by the females from their diet and may be less affected (Wu, 2009). In oviparous species, the egg contains all of the nutrients required by the embryo for successful development (Congdon *et al.,* 1983). Mothers allocate available nutrients directly from food consumed; from storage in the liver or fat deposits; and from storage of egg yolk during vitellogenesis, protein to the albumen layer and calcium to the eggshell (Van Dyke and Griffith, 2018).

We determined how reproduction by female Murray River short-necked turtles (*Emydura macquarii*) is affected by turtle diet composition (i.e. relative abundances of dietary plant and animal species) by comparing differences in maternal diet and allocation of resources to reproduction among females at four wetlands in north-central Victoria, Australia. We used *E. macquarii* as a model species because it is a generalist consumer that is able to change its diet as food availability changes (Chessman, 1986; Spencer *et al.,* 2014; Petrov *et al.,* 2020). We have previously found that *E. macquarii* diets differ among the four abovementioned sites and that the sites vary in the availability and accessibility of prey (Petrov *et al.,* 2018; Petrov *et al.,* 2020). *Emydura macquarii* is also important as a model species because they are listed as vulnerable in Victoria (Van Dyke *et al.,* 2018) and have declined by as much as 67% across the Murray River catchment (Chessman, 2011; Van Dyke *et al.,* 2019). Populations of *E. macquarii* in the Murray River catchment are heavily biased towards older individuals, particularly females, with juveniles being uncommon. A lack of juveniles suggests low rates of recruitment, potentially owing to high rates of nest predation by the European red fox *Vulpes vulpes* (Thompson, 1993; Van Dyke *et al.,* 2019). Additionally, the availability of food (i.e. relative abundances of dietary plant and animal species) and its effects on reproduction has not previously been studied as a factor in their decline.

Specifically, we tested for differences in total clutch mass, individual egg mass and hatching success across the four wetlands. Because maternal diet constrains the nutrients available to be allocated to each egg (Congdon *et al.,* 1983; Finkler and Claussen, 1997), we also compared the bulk composition of eggs from females from each wetland. Specifically, we determined the amount of water, total protein, total lipid and energy allocated to each egg. We also repeated our prior stable isotope comparison (Petrov *et al.,* 2020) to verify that the turtles in these wetlands still exhibited the same dietary differences. We predicted that females from Longmore Lagoon (low filamentous algae availability, high carnivory, empty stomachs) would exhibit reduced reproductive output, such that clutch mass and individual egg mass are reduced, compared to females from Safes Lagoon (high filamentous algae availability, high herbivory, no empty stomachs), and that females from Cockatoo Lagoon and Gunbower Creek (intermediate food availability) would exhibit some intermediate values for the above parameters (Petrov *et al.,* 2020). Lastly, we compared hatching success across sites to determine whether any differences we detected in egg composition were associated with developmental success.

## Materials and Methods

### Study sites

This study was conducted at four wetlands adjacent to the Murray River between Cohuna and Gunbower, Victoria, Australia: the wide-bodied oxbow lakes Cockatoo Lagoon (35.919, 144.360), Longmore Lagoon (−35.963, 144.393) and Safes Lagoon (−35.687, 144.156), which are connected to Gunbower Creek (−35.861, 144.331) by a regulated network of channels and pipes (Petrov *et al.,* 2018). Our previous research found *E. macquarii* at Safes Lagoon consume large amounts of filamentous green algae, whereas *E. macquarii* at Longmore Lagoon are more carnivorous (Petrov *et al.,* 2018; Petrov *et al.,* 2020). At Longmore Lagoon, more turtles had empty stomachs and female turtles had lower body condition (Petrov *et al.,* 2020), both potential consequences of reduced food availability. At Cockatoo Lagoon and Gunbower Creek, *E. macquarii* are omnivorous (Petrov *et al.,* 2018). These dietary differences were verified using both stomach contents and stable isotope analyses and were driven by differences in local food availability (Petrov *et al.,* 2018; Petrov *et al.,* 2020). Although *E. macquarii* is likely capable of moving between at least some of the four wetlands, the isotopic differences we previously reported (Petrov *et al.,* 2020) suggest either that such movements are rare or that turtles forage primarily in only one area for prolonged periods of time.

### Turtle sampling and egg collection

*Emydura macquarii* were trapped during Austral Spring between 30 October 2016 and 6 November 2016, using baited cathedral traps. Traps were set at least 5 m apart and were checked every 10–14 hr (Petrov *et al.,* 2020). Trapping continued until ~10 gravid female *E. macquarii,* as determined by palpation*,* were sampled from each site. Gravid females that were not ready to lay, as indicated by the presence of soft eggs relatively anterior in the body cavity, were released with non-gravid females. Gravid females were weighed (to the nearest grams, using scales up to 5 kg) and measured using large (up to 102 cm) callipers (carapace and plastron length and width, to the nearest millimeter).

The gravid females were held on-site in water-filled tubs and injected intramuscularly with 20 mg/kg of oxytocin to induce oviposition (Ewert and Legler, 1978); turtles were re-injected every 48 hr until no eggs were felt via palpation, but only one injection was necessary for the majority of turtles. The number of eggs laid by each female and the weight (to the nearest grams measured with digital scales), length and width (to the nearest millimeters, measured with vernier callipers) of each egg were recorded (see Supplementary Material, Tables S6–S9). The first and last eggs of each clutch were frozen and stored at −20°C for isotopic and compositional analysis. After oviposition, females were reweighed. Claw clippings were sampled from each of the toes of the left hind leg of each turtle for stable isotope analysis (Petrov *et al.,* 2020). Claw clippings reflect the isotopic composition of the food eaten over the past *~*12 months (Seminoff *et al.,* 2007). Claw samples were frozen and stored at −20°C for stable isotope analysis.

The remaining eggs in each clutch were labelled with a pencil to identify site, maternal identity and laying order within the clutch. The eggs were placed in moist vermiculite and transported to Western Sydney University for incubation. Each clutch was divided in half into one of two incubation temperatures, with ‘even numbered’ eggs incubated at 26°C and ‘odd numbered eggs’ incubated at 30°C. These incubation temperatures were used for another study concurrent with this project. The two treatments were incubated in separate incubators. Within both incubators, eggs were grouped by clutch and placed in covered (but unsealed) plastic tubs filled with a 1:1 mixture, as determined by weight, of vermiculite and deionized water. The mass of each tub was measured weekly, and any mass lost from evaporation of water was replaced with water from a spray bottle to maintain the water content of the vermiculite in each container (Packard *et al.,* 1987). Tubs were randomly rotated around the incubator to account for shelf-specific variations in temperature. Eggs that did not develop or developed fungi were removed from the clutch and frozen at −20°C. We recorded the duration of incubation in days. Upon hatching, the hatchlings were weighed (to the nearest grams with a digital scale) and measured (carapace and plastron length and width, to the nearest millimeters, with vernier callipers) for a concurrent study. The number of successful hatchings was recorded. This work was conducted under Western Sydney University Animal Ethics Committee Animal Research Authority A11794. This work was conducted under DEWLP permit 10 008 041 and DEPI permit RP1225.

### Compositional analysis of eggs

All frozen eggs samples and maternal claw clippings were freeze dried at −40°C to asymptotic mass using an Edwards Modulyo Freeze Dryer (Burgess Hill, United Kingdom). Eggs (including the shell) and claw clippings were homogenized to powder using a Retsch 400MM ball mill (Haan, Germany) and stored in a desiccator until further analysis. We estimated the moisture, protein, lipid and energy contents of each homogenized egg (i.e. two eggs per clutch, *n* = 62). We determined the water content (g) of each egg by subtracting the dry mass of the egg from the initial wet egg mass, when it was first laid. Total protein content was determined by measuring the total nitrogen content of each sample using a Thermo Scientific Delta V Advantage isotope ratio mass spectrometer (Waltham, MA, USA) at the Centre for Carbon, Water and Food of the University of Sydney, following Van Dyke *et al.* (2014). As per the stable isotope analyses below, 1 mg of egg homogenate was placed into tin capsules and packaged into 96-well microplates prior to analysis. The nitrogen content (%) of each sample was multiplied by 6.25, following the Dumas method (Jung *et al.,* 2003) to estimate the protein content (%) of each sample. Percentage protein was then multiplied by the dry mass of the sample to determine total bulk protein mass (g). Total lipid was determined by a two-step extraction and separation process. Lipids were extracted from a subsample of homogenate (*~*0.5 mg) via further homogenization in a chloroform–methanol–water (1:1:1) mix using a Thomas Scientific PYREX dounce homogenizer tissue grinder. The subsample homogenate was filtered through a Büchner funnel and transferred to a 50-ml graduated cylinder for lipid separation. The chloroform/lipid layer of the filtrate was allowed to separate from the methanol/water layer for *~*10 min, and the methanol/water layer was removed via aspiration (Van Dyke *et al.,* 2014). The remaining chloroform was evaporated using nitrogen gas. Total lipid was determined gravimetrically by dividing the mass of the extracted lipid by the mass of the subsample to calculate the proportion of the lipid relative to the total subsample mass. This was then multiplied by the total dry mass of the whole egg, giving the amount of lipid (g) in the entire egg. Total energy (kJ) was determined using a Parr 6200 calorimeter (Parr Instrument Company, Illinois, USA). A subset of each sample homogenate (*~*0.4 mg) was pressed into pellets, weighed and combusted in the calorimeter (Thompson *et al.,* 1999). The calorimeter was calibrated every 20 samples using benzoic acid.

### Stable isotope analysis

Stable isotope analyses were used to validate the site differences in adult turtle diet found in Petrov *et al.* (2020) and determine whether the isotopic compositions (δ^13^C and δ^15^N) of the eggs reflected the isotopic compositions of their mothers. We analysed δ^13^C and δ^15^N of the claw clippings sampled from each gravid *E. macquarii* and δ^13^C and δ^15^N of the eggs. One milligram of claw homogenate and 1 mg of egg homogenate were weighed into tin capsules and packaged into 96-well microplates prior to analysis. δ^13^C and δ^15^N were determined using a Delta V Advantage isotope ratio mass spectrometer (Thermofisher Scientific, Waltham, MA, USA) coupled to a ConfloIV and FlashHT at the Centre for Carbon, Water and Food of the University of Sydney. For full isotope ratio mass spectrometer methodology, refer to Petrov *et al.* (2020). Isotopic values are presented in delta notation (‰), relative to Vienna Pee Dee Belemnite (VPDB) for carbon and ambient air for nitrogen. Precision was between 0.03 and 0.05‰ for carbon analysis (1 SD, *n* = 2) and between 0.02 and 0.04‰ for nitrogen analysis (1 SD, *n* = 2).

### Statistical analyses

#### Comparing isotopic composition of female *E. macquarii* across sites

We tested whether female *E. macquarii* differed in isotopic composition (δ^13^C and δ^15^N) among sites, to confirm that the adult turtle differences in isotopic composition reported in Petrov *et al.* (2020) were still present. We ran a multivariate analysis of covariance (MANCOVA) in SAS (PROC GLM) comparing the isotopic composition, δ^13^C and δ^15^N of *E. macquarii* females among sites. We set δ^13^C and δ^15^N as the dependent variables, site as the fixed main effect, maternal body size [straight carapace length (SCL)] as a covariate and the interaction of site and maternal body size as the effect of interest.

#### Effect of maternal diet on reproductive allocation

To determine the effect of maternal diet on reproductive allocation, we used wetland as a proxy for diet based on the among-wetland differences in diet we had previously reported (Petrov *et al.,* 2020). We compared the following reproductive parameters across the four wetlands: total clutch mass (as an index of clutch size), individual egg mass and bulk egg composition (including water, protein, lipid, energy and isotopic composition). We also tested for among-site differences in hatching success to determine whether any egg composition differences we detected were related to differences in hatching success. To test for among-site differences in total clutch mass, we ran an analysis of covariance (ANCOVA) in SAS (PROC GLM) with log-transformed total clutch mass as the response variable, site as a fixed effect and log-transformed maternal body size (SCL) as a covariate.

To test for differences in individual egg mass, we ran an ANCOVA in SAS (PROC MIXED) with log-transformed egg wet mass as the response variable, site and laying order as fixed effects, log-transformed maternal body size as a covariate and maternal ID as a random effect. Laying order was included in the analysis as an additional covariate to test for potential egg size differences between eggs laid early in a clutch and those laid later (Loudon, 2014). Maternal ID was included as a random effect in the model to account for pseudoreplication of the clutches, because we included two eggs per clutch (Hurlbert, 1984). Maternal body size was included in the analysis of total clutch mass and individual egg mass because the volume of the mothers’ body cavities and diameters of their pelvic openings may influence both clutch and egg width (Congdon, 1989).

To compare the moisture, protein, lipid and energy contents of each egg, we ran four separate ANCOVAs in SAS (PROC MIXED). The PROC MIXED procedure allows random variation to be modelled at both within- and between-subject levels concurrently. For each separate analysis, we log-transformed moisture, protein, lipid and energy contents to meet the assumptions of an ANCOVA as evaluated by examination of residuals. Log-transformed moisture, protein, lipid and energy were set as the response variables in each analysis. In each analysis, site and laying order were included as fixed effects, log-transformed egg dry mass as a covariate and maternal ID as a random effect. To determine whether the isotopic compositions (δ^13^C and δ^15^N) of the eggs reflect the isotopic compositions of their mothers, we ran two separate ANCOVAs in SAS (PROC MIXED), one each for δ^13^C and δ^15^N. We set the isotopic composition of the eggs (δ^13^C or δ^15^N) as the response variable, the isotopic composition of the mothers (δ^13^C or δ^15^N) and site as fixed effects and maternal ID as a random effect.

#### Hatching success

To determine the effect of maternal diet on hatching success, we ran a generalized linear mixed model in SAS (PROC GLIMMIX). We set the response variable as the number of eggs that hatched divided by the number of eggs laid, with site and temperature as fixed effects and maternal ID as a random factor. Incubation temperature was included because it can affect embryonic physiology in reptiles (Deeming and Ferguson, 1991; Booth, 1998; Dormer *et al.,* 2016).

In all ANCOVAs, we tested full factorial designs initially. When interactions were not statistically significant, their variance component was rolled into the error term. We report the full models in supplementary materials. Normality and homoscedasticity were assessed for all statistical tests by examining the plots of residuals graphically, in conjunction with the Shapiro–Wilk test (*P* > 0.05). All statistical tests were assessed at *α* = 0.05. Best-fit covariate structures for random effects were determined using Akaike's Information Criterion (AIC) in mixed models. Where there were more than two levels of a statistically significant main effect of interest, the significance was further examined using post hoc Tukey–Kramer multiple comparisons.

## Results

Thirty-one gravid female *E. macquarii* were collected from the four wetlands (nine from Safes Lagoon, seven from Cockatoo Lagoon, eight from Longmore and seven from Gunbower Creek).

### Isotopic composition of *E. macquarii* across sites

In the MANCOVA of maternal δ^15^N and maternal δ^13^C against site and maternal body size, no interaction terms were statistically significant (Supplementary Material, Table S1); their sums of squares were incorporated into the error term. Maternal δ^15^N and maternal δ^13^C differed significantly across sites (Supplementary Material, Table S2). Univariate analyses of maternal δ^15^N and maternal δ^13^C showed that the multivariate differences in site occurred for both maternal δ^15^N and maternal δ^13^C (Supplementary Material, Tables S3 and S4). *Emydura macquarii* from Cockatoo Lagoon were significantly lower in δ^13^C compared to *E. macquarii* from Gunbower Creek (*P* < 0.002), Longmore Lagoon (*P* < 0.001) and Safes Lagoon (*P* < 0.001; Supplementary Material, Table S5). *Emydura macquarii* from Safes Lagoon were significantly higher in δ^13^C compared to *E. macquarii* from Gunbower Creek (*P* < 0.003; Supplementary Material, Table S5). *Emydura macquarii* from Safes Lagoon had significantly lower δ^15^N compared to *E. macquarii* from Cockatoo Lagoon (*P* < 0.003), Gunbower Creek (*P* < 0.002) and Longmore Lagoon (*P* < 0.001; Supplementary Material, Table S5). *Emydura macquarii* from Longmore Lagoon had similar δ^15^N compared to Cockatoo Lagoon and Gunbower Creek (all *P* > 0.077). The pairwise among-site differences for Safes Lagoon, Cockatoo Lagoon and Gunbower Creek aligned with the site differences in adult turtle diet found by Petrov *et al.* (2018, 2020) (Supplementary Material, Table S5 and Fig. S1), whereby *E. macquarii* from Safes Lagoon had a more herbivorous diet and Cockatoo Lagoon and Gunbower Creek exhibited an omnivorous diet. While *E. macquarii* from Longmore Lagoon were still high in δ^15^N in this study (Supplementary Material, Table S5), the difference was no longer significant, as reported in 2020.

**Table 1:** ANCOVA results comparing the relationships between total clutch mass, across sites and log-transformed maternal body size (SCL)

Effect	Num *df*	Den *df*	*F*	*P*
Site^*^	3	33	3.20	0.040
Maternal body size^*^	1	33	41.27	<0.001
Maternal body size × site^*^	3	33	3.25	0.038

Asterisks indicate significant effects.

### Effect of maternal diet on reproductive allocation

#### Total clutch mass

**Figure 1:**
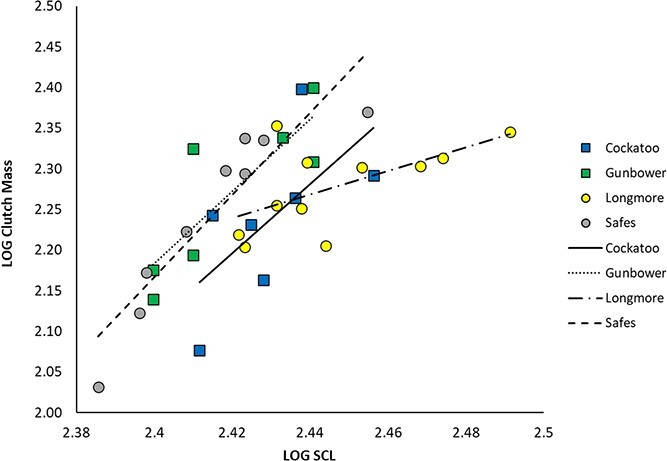
Relationships between total clutch mass and maternal body size, compared across the algae-rich site, Safes Lagoon; algae-poor site, Longmore Lagoon; and intermediate sites, Cockatoo Lagoon and Gunbower Creek.

The interaction between site and maternal body size on total clutch mass was significant (*P* < 0.038; Table 1). Average total clutch mass was 177.1 ± 41.04 g (range, 119.3–250.1 g) at Cockatoo Lagoon, 189.7 ± 42.0 g (range, 137.9–250.8 g) at Gunbower Creek, 191.0 ± 23.5 g (range, 159.9–225.4 g) at Longmore Lagoon and 180.0 ± 43.3 g (range, 107.5–217.8 g) at Safes Lagoon (Supplementary Material, Tables S6–S9). There was a positive relationship between total clutch mass and maternal body size at each site (Fig. 1; Supplementary Material, Table S10). At Longmore Lagoon, the slope of the relationship between maternal body size and total clutch mass is significantly smaller than at the other three sites (Fig. 1; Supplementary Material, Table S10). Thus, clutch size of Longmore turtles increased at a slower rate relative to body size than it did in turtles from the other three sites (Fig. 1). Also, at all body sizes, *E. macquarii* at Cockatoo Lagoon produced significantly smaller clutches in comparison to *E. macquarii* at Safes Lagoon and Gunbower Creek, though the magnitude of the difference was slight (Fig. 1; Supplementary Material, Table S10).

**Table 2:** ANCOVA comparing the relationships between individual egg mass, across sites, order and log-transformed maternal body size (SCL)

Effect	Num *df*	Den *df*	*F*	*P*
Site	3	531	2.10	0.099
Order*	1	531	6.82	0.009
Order × site	3	531	2.62	0.050
Maternal body size	1	531	0.15	0.694
Maternal body size × site	3	531	2.10	0.100
Order × maternal body size^*^	1	531	6.64	0.010
Order × maternal body size × site	3	531	2.62	0.050

Asterisks indicate significant effects.

#### Egg mass

Individual egg mass did not differ across sites (*P* = 0.099; Table 2). Individual egg mass was influenced by an interaction between laying order and maternal body size (SCL, *P* < 0.010). The interactions of order and site and order, maternal body size and site were borderline (all *P* > 0.050), and there were no other significant interactions (Table 2). To determine how egg size is affected by the interaction of laying order and maternal body size, we examined the slopes of the linear relationship between egg mass, laying order and maternal body size (Table 3). Smaller turtles tended to produce larger eggs later in a clutch, whereas larger turtles tended to produce larger eggs earlier in a clutch. The interaction between laying order and maternal SCL is statistically significant, but the effect is small (Table 3).

**Table 3:** Parameter estimates (±SE) of the relationships between individual egg mass, laying order and log-transformed maternal body size (SCL)

Effect	Estimate
Intercept	−0.419 ± 0.786
Order	0.031 ± 0.016
Maternal body size	0.582 ± 0.323
Order × maternal body size	−0.012 ± 0.006

#### Egg composition

In the four ANCOVAs of egg moisture, protein, lipid and energy compared across site, laying order, egg dry mass and maternal ID, no interactions were statistically significant (Supplementary Material, Tables S11–S14); their sums of squares were incorporated into the error term. The moisture content (*P* < 0.001), protein content (*P* < 0.001), lipid content (*P* = 0.001) and energy content (*P* < 0.001) of the eggs differed significantly with egg dry mass but did not differ significantly with site (all *P* > 0.154) or laying order (all *P* > 0.160; Supplementary Material, Tables S15–S18). Thus, egg compositions did not differ across sites in this study.

Since there were no differences in egg composition in our study, the average egg wet mass was 9.98 ± 0.16 g, and the average water fraction was 78.41 ± 0.30%. After removing water, the average egg lipid content was 14.52 ± 0.64% dry mass, and the average protein content was 43.42 ± 0.72% dry mass. The average energy content was 23.29 ± 0.21 kJ/mg dry mass. More compositional details are available in the Supplementary Material (Tables S6–S9).

**Figure 2:**
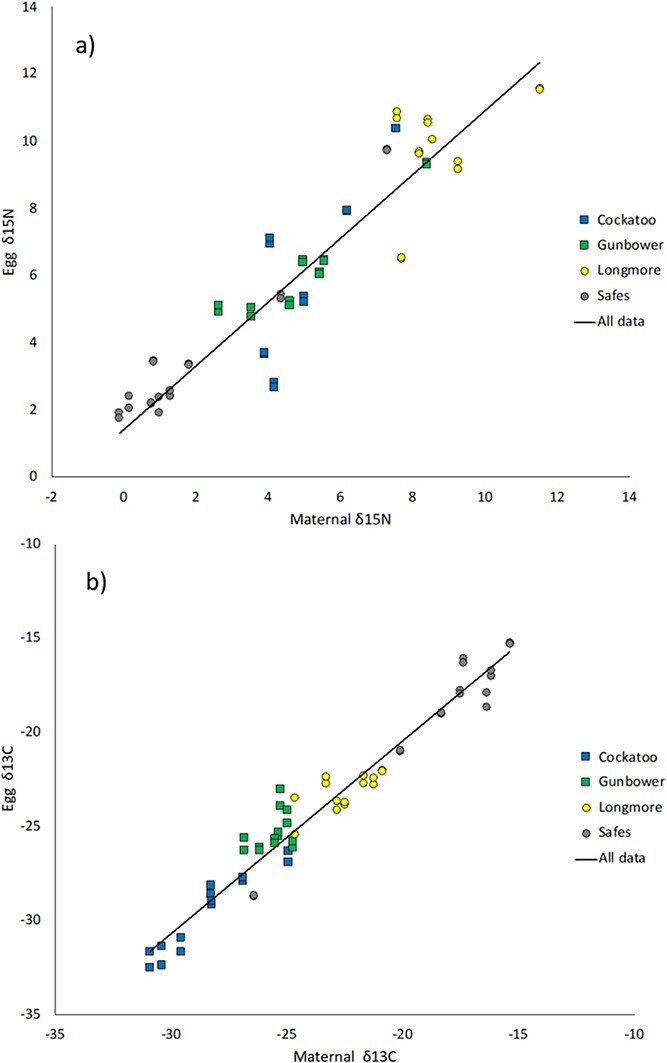
(a) The relationship between egg δ^15^N and maternal δ^15^N. The slope of the relationship between egg δ^15^N and maternal δ^15^N suggests mothers are allocating the same ratios of heavy and light nitrogen into their eggs as they themselves have. (b) The relationship between egg δ^13^C and maternal δ^13^C. The slope of the relationship between egg δ^13^C and maternal δ^13^C suggests that mothers allocate similar ratios of heavy and light carbon to their eggs as they have in their bodies.

”Egg δ^15^N was influenced by maternal δ^15^N (*P* < 0.001); however, there were no other significant interactions (Table 4). To determine how egg δ^15^N is affected by maternal δ^15^N, we examined the slope of the linear relationship between egg δ^15^N and maternal δ^15^N, which revealed a significant positive relationship between egg δ^15^N and maternal δ^15^N (Fig. 2a, Table 5). The slope of the relationship between egg δ^15^N and maternal δ^15^N was very close to one, and the y-intercept was approximately 1 (Fig. 2a, Table 5), suggesting mothers allocate slightly more ^15^N than ^14^N to their eggs, but at a rate that is not influenced by their own δ^15^N. Similarly, egg δ^13^C was influenced by maternal δ^13^C (*P* < 0.001); however, there were no other significant interactions (Table 4). To determine how egg δ^13^C is affected by maternal δ^13^C, we examined the slope of the linear relationship between egg δ^13^C and maternal δ^13^C, which revealed a significant positive relationship between egg δ^13^C and maternal δ^13^C (Fig. 2b and Table 5). The slope of the relationship between egg δ^13^C and maternal δ^13^C was just slightly less than 1, and the y-intercept was not different from zero (Fig. 2b; Table 5), suggesting mothers are allocating similar ratios of heavy and light carbon into their eggs as they themselves have. The very slight depletion in the slope suggests that at most, mothers are allocating slightly less carbon ^13^C to their eggs than carbon ^12^C.

**Table 4:** ANCOVA comparing the relationship between the isotopic composition of the eggs (δ^15^N and δ^13^C) with the isotopic composition of the mothers (δ^15^N and δ^13^C), across sites

	Num *df*	Den *df*	*F*	*P*
δ^15^N				
Maternal δ^15^N^*^	1	30	53.06	<0.001
Site	3	30	2.83	0.055
Maternal δ^15^N × site	3	30	2.39	0.089
**δ** ^**13**^ **C**				
Maternal δ^13^C*	1	29	35.88	<0.001
Site	3	29	2.16	0.114
Maternal δ^13^C × site	3	29	2.37	0.091

Asterisks indicate significant effects.

#### Hatching success

Hatchlings emerged after ~62 ± 2.5 and 52 ± 2.3 d for 26 and 30°C incubation temperatures, respectively. Hatching success rates did not differ across sites (*P* = 0.121) or incubation temperatures (*P* = 0.840), and there were no significant interactions between site and temperature (*P* = 0.332; Supplementary Material, Table S19). At 26°C, the proportion of eggs that hatched was 0.79 ± 0.28 at Cockatoo Lagoon, 0.98 ± 0.06 at Gunbower Creek, 0.72 ± 0.30 at Longmore Lagoon and 0.96 ± 0.11 at Safes Lagoon. At 30°C, the proportion of eggs that hatched was 0.82 ± 0.33 at Cockatoo Lagoon, 0.95 ± 0.08 at Gunbower Creek, 0.80 ± 0.38 at Longmore Lagoon and 0.91 ± 0.08 at Safes Lagoon.

## Discussion

Maternal diet influences how energy and nutrients are allocated to offspring during reproduction. We found a positive relationship between clutch mass and maternal body size in turtles from each site, with clutch mass increasing with maternal body size. This finding was expected, as an increase in clutch mass with increasing maternal body size is common across oviparous species (Gatto *et al.,* 2020). At Longmore Lagoon, where food is limited (Petrov *et al.,* 2018), clutch size increased at a slower rate relative to body size than at the other three sites, suggesting a potential constraint of maternal diet on reproductive allocation. Our previous studies found female *E. macquarii* at Longmore Lagoon tend towards carnivory and have higher rates of empty stomachs and lower body condition (Petrov *et al.,* 2018; Petrov *et al.,* 2020). Although our analysis found that δ^15^N of *E. macquarii* from Longmore Lagoon in this study was no longer significantly different to δ^15^N of *E. macquarii* from the intermediate sites of Cockatoo Lagoon or Gunbower Creek, individual *E. macquarii* from Longmore Lagoon tended to have the highest values in the study, suggesting some individuals may be trending towards carnivory, as previously reported. In general, there may be greater herbivory occurring now at Longmore than we observed in the prior study. We previously suggested that the scarcity of food at Longmore Lagoon drove the reduced body condition we detected in *E. macquarii* (Petrov *et al.,* 2020). Here, our data further suggest that reduced food abundance also drives reductions in the number of eggs that a female can produce. Future studies should experimentally manipulate the amount of food (especially filamentous green algae) available to *E. macquarii* to confirm the correlations we have observed between algae availability and the number of eggs a female produces. Likewise, laboratory-based studies that identify how physiological allocation decisions (Dunham *et al.,* 1989) change, which result in the patterns we report here, would be excellent in order to fully understand the mechanisms underlying the shifts in fecundity that we observed.

Our results are similar to those previously reported in other reptiles. For example, adult female brown anole lizards (*Anolis sagrei*) raised under low prey availability produced significantly fewer eggs per clutch than females raised under high prey availability (Warner *et al.,* 2015). A similar trend has also been observed in reproductive female phrynosomatid lizards (*Sceloporus virgatus*), with females producing smaller clutch sizes when prey availability and rainfall are low (Abell, 1999). Clutch size in turtles is likely determined by maternal body condition and the availability of prey in late summer and autumn, as follicles begin to enlarge and continue to mature through to winter (Harless and Morlock, 1979; Georges, 1983; Wilkinson and Gibbons, 2005). As such, the availability of prey at this time likely influences the amount of energy available for reproduction.

Unexpectedly, female *E. macquarii* at Cockatoo Lagoon produced slightly smaller clutches in comparison to *E. macquarii* at Safes Lagoon and Gunbower Creek. *Emydura macquarii* at Cockatoo Lagoon have similar diets to *E. macquarii* at Gunbower Creek (Petrov *et al.,* 2020), with both sites having similar levels of turbidity and filamentous green algae abundance (Petrov *et al.,* 2018). Because of the similarities between the environment and diets of *E. macquarii* at Cockatoo Lagoon and Gunbower Creek, it was expected that any effect of maternal diet observed at one of these sites would likely be present at the other. However, no reduction in clutch size at Gunbower Creek in comparison with Cockatoo Lagoon was detected. The smaller clutch sizes observed at Cockatoo Lagoon may be among-year variations in maternal diet that our stable isotope approach was not able to detect. Environmental sampling and isotopic sampling by Petrov *et al.* (2018 and 2020) were conducted in 2015 and the start of 2016, while the reproductive allocation data in the present study were collected at the end of 2016. If prey availability or abundance at Cockatoo Lagoon declined early in 2016, at the time when follicles begin to enlarge (Harless and Morlock, 1979; Georges, 1983), *E. macquarii* females may have had less energy available for reproduction than *E. macquarii* at Gunbower Creek. Differences in prey availability or abundance could have also occurred at the other sites; however, this was not detected through the reproductive allocation data. As such, future studies examining reproductive allocation should sample the environment and diet (i.e. stomach content and isotopic sampling) throughout reproduction.

**Table 5:** Parameter estimates (±SE) for the relationships between (i) egg δ^15^N and maternal δ^15^N, and (ii) egg δ^13^C and maternal δ^13^C and associated *P* values

	Estimate	y-intercept	*P* value
δ^15^N	1.029 ± 0.171	1.675 ± 0.507	<0.001
δ^13^C	1.172 ± 0.086	2.548 ± 1.597	<0.001

We found no significant effect of maternal diet on egg mass, with female *E. macquarii* producing similar-sized eggs across sites. This result suggests that females alter the number of eggs produced in response to prey availability but do not alter egg size. This result is similar to the findings of Warner *et al.* (2015) who found that female brown anole lizards *A. sagrei* raised on a low prey diet produced similar-sized eggs to females on a high prey diet. Egg size in the current study did however vary with an interaction of laying order and maternal body size (Congdon and Gibbons, 1985; Congdon and Gibbons, 1987). Freshwater turtles construct nest chambers shaped like a flask, with the bottom of the nest being wider in diameter than the top (Goode, 1965). The wider base allows more eggs to be incubated at the bottom of the nest (Booth, 2010), away from daily temperature fluctuations (Thompson, 1988). Smaller turtles may produce smaller eggs earlier in a clutch to ensure more eggs are protected against temperature fluctuations. Laying bigger eggs at the bottom of a nest has also been hypothesized to support the mass of the eggs at the top of the nest during development (Tucker and Janzen, 1998); however, this hypothesis and reasons for bigger eggs being laid later in a clutch are yet to be explored.

We found no effect of site on the compositions of eggs (i.e. moisture, protein, lipid or energy), suggesting *E. macquarii* at different sites are allocating similar proportions of nutrients into their eggs regardless of maternal diet. However, this study does not take into account the proportions of inorganic ions such as calcium, sodium, magnesium or iron, which constitute 5% of an eggs composition (Thompson *et al.,* 2000), or the proportions of specific amino or fatty acids (Speake and Thompson, 1999; Thompson *et al.,* 2000). Elevated levels of omega-3 fatty acids in particular increased cognitive maturation in ring-billed gulls (*Larus delawarensis*), which led to earlier fledging (Lamarre *et al.,* 2021). Further research should examine the proportion of these additional macro- and micronutrients in eggs to determine whether maternal diet is influencing egg composition and potentially hatchling size and physiology at a scale not studied here.

Female turtles allocated similar ratios of maternal δ^15^N and maternal δ^13^C to their eggs as they had available in their bodies. Offspring are expected to have δ^15^N values that are approximately one trophic position higher than their mother, as offspring are consuming resources that are derived from their mother (Jenkins *et al.,* 2001), and some of this difference may occur at the maternal allocation step. Our analysis found that females were allocating slightly more ^15^N to their eggs, enough to increase the δ^15^N by about 1. This is less than a full trophic level increase (Post, 2002) but still indicates a slight enrichment. Females were also found to allocate similar ratios of heavy and light carbon into their eggs, as found in their own bodies.

The correlation observed between maternal δ^15^N and δ^13^C and egg isotopic δ^15^N and δ^13^C was similar to those found in other studies of turtles (Caut *et al.,* 2008; Zbinden *et al.,* 2011; Frankel *et al.,* 2012; Carpentier *et al.,* 2015). For instance, in leatherback turtles (*Dermochelys coriacea*), a positive relationship was found between maternal blood δ^15^N and δ^13^C and egg yolk δ^15^N and δ^13^C (Caut *et al.,* 2008), while in loggerhead turtles (*Caretta caretta*), a significant relationship was found between egg yolk δ^15^N and δ^13^C and respective adult tissues (Carpentier *et al.,* 2015). Whole blood of *C. caretta* was the best predictor for egg yolk isotopic values, with loggerhead turtle egg yolk enriching in δ^15^N by ~1 trophic position (Carpentier *et al.,* 2015), as observed in the current study. A distinguishing difference between our study and those mentioned above is the samples used. In the current study, we analysed δ^15^N and δ^13^C from whole turtle eggs and maternal claws, whereas other studies have isolated egg yolk and compared this to maternal blood (Caut *et al.,* 2008; Carpentier *et al.,* 2015) and maternal carapace (Zbinden *et al.,* 2011). Despite these sampling differences, we observed similar relationships between egg isotopic values and maternal isotopic values.

Taken together, these results have long-term implications for the viability and conservation of turtle populations. *Emydura macquarii* is in decline in the Murray River system, at least in part due to high nest predation by European red foxes *V. vulpes* (Thompson, 1993; Van Dyke *et al.,* 2019). The reduction in clutch size found in *E. macquarii* in response to food limitations may further contribute to the species decline, with potentially reduced recruitment across years (Dimond and Armstrong, 2007; Vincenzi *et al.,* 2008; Spencer, 2018). Our prior work demonstrated that the food abundance variations we tested here were associated with increased turbidity and a scarcity of primary producers (Petrov *et al.,* 2018). As much of the Murray River system is degraded (Quiggin, 2001), similar to Longmore Lagoon (Petrov *et al.,* 2018), food scarcity may be a common factor impacting *E. macquarii* across its range. If food scarcity is common across the region, then its impacts on fecundity may combine with the impacts of invasive fox predation on nests. Thus, as foxes reduce the total number of nests that survive to hatching, food scarcity may, in addition, reduce the number of eggs that are present in each nest and compound reductions in *E. macquarii* recruitment. Management that aims to increase turtle recruitment may need to focus on improving wetland food abundance in addition to reducing the threat of foxes. If current trends continue, it may become increasingly important to supplement the populations via head starting to potentially bypass reproductive constraints (Spencer *et al.,* 2017) and prevent further species declines. Additional research is needed to determine how *E. macquarii* diets vary across the river system and also to determine the impacts of food limitations on two sympatric, declining turtle species, the Eastern long-necked turtle (*Chelodina longicollis*) and the broad-shelled turtle (*Chelodina expansa*)*.*

## Supplementary Material

Web_Material_coae033

## Data Availability

The dataset supporting the results of this article are available at https://doi.org/10.5061/dryad.05qfttfbg.
