# Supplementary material for: Maternal diet influences fecundity in a freshwater turtle undergoing population decline
Source: Conserv Physiol. 2024 May 27;12(1):coae033. doi: 10.1093/conphys/coae033 (PMC11129712; doi:10.1093/conphys/coae033)
Supplement: Web_Material_coae033 [file web_material_coae033.pdf]

Maternal diet influence fecundity in a freshwater turtle undergoing population decline

Kristen Petrov[1], James U. Van Dyke[2*], Arthur Georges[3], Claudia Keitel[4], Ricky-John Spencer[1]

1. School of Science, Hawkesbury Institute, Western Sydney University Locked Bag, 1797, Penrith South DC NSW 2751, Australia.
2. School of Agriculture, Biomedicine and Environment, La Trobe University, Albury-Wodonga Campus, PO Box 821, Wodonga, VIC 3689 Australia
3. Institute for Applied Ecology, University of Canberra, ACT 2601, Australia
4. School of Life and Environmental Sciences, University of Sydney, 380 Werombi Road, Brownlow Hill, NSW 2570, Australia.

*Corresponding author:

email: j.vandyke@latrobe.edu.au

phone: +61 260249712

Supplementary material:

Table S1. Results of MANCOVA analysis of differences in maternal δ^{15}N and maternal δ^{13}C across sites and maternal body size.

Effect	Pillai's Trace	*F*	Num *df*	Den *df*	*P*
Site	0.327	1.50	6	46	0.2002
Maternal body size	0.149	1.92	2	22	0.1703
Site x Maternal body size	0.318	1.45	6	46	0.2162

Table S2. Results of MANCOVA analysis of differences in maternal δ^{15}N and maternal δ^{13}C across sites and maternal body size, without the interaction of site and maternal body size.

Effect	Pillai's Trace	*F*	Num *df*	Den *df*	*P*
Site	1.306	16.22	6	52	<0.001
Maternal body size	0.056	0.74	2	25	0.4880

Table S3. Univariate effects of site and maternal body size on maternal δ^{15}N.

Effect	*Num DF*	*Den DF*	*F value*	*P*
Site	3	30	13.36	<0.001
Maternal body size	1	30	0.02	0.9026

Table S4. Univariate effects of site and maternal body size on maternal δ^{13}C.

Effect	*Num F*	*Den DF*	*F value*	*P*
Site	3	30	23.72	<0.001
Maternal body size	1	30	1.39	0.2486

Table S5. Least square means Tukey-Kramer post-hoc analysis showing site differences in maternal δ^{15}N and maternal δ^{13}C.

Maternal δ^{15}N	*P* values	Maternal δ^{13}C	*P* values
Cockatoo – Gunbower	1.000	Cockatoo – Gunbower	0.002
Cockatoo – Longmore	0.077	Cockatoo – Longmore	< 0.001
Cockatoo - Safes	0.003	Cockatoo - Safes	< 0.001
Gunbower - Longmore	0.103	Gunbower - Longmore	0.493
Gunbower – Safes	0.002	Gunbower – Safes	0.003
Longmore – Safes	< 0.001	Longmore – Safes	0.195

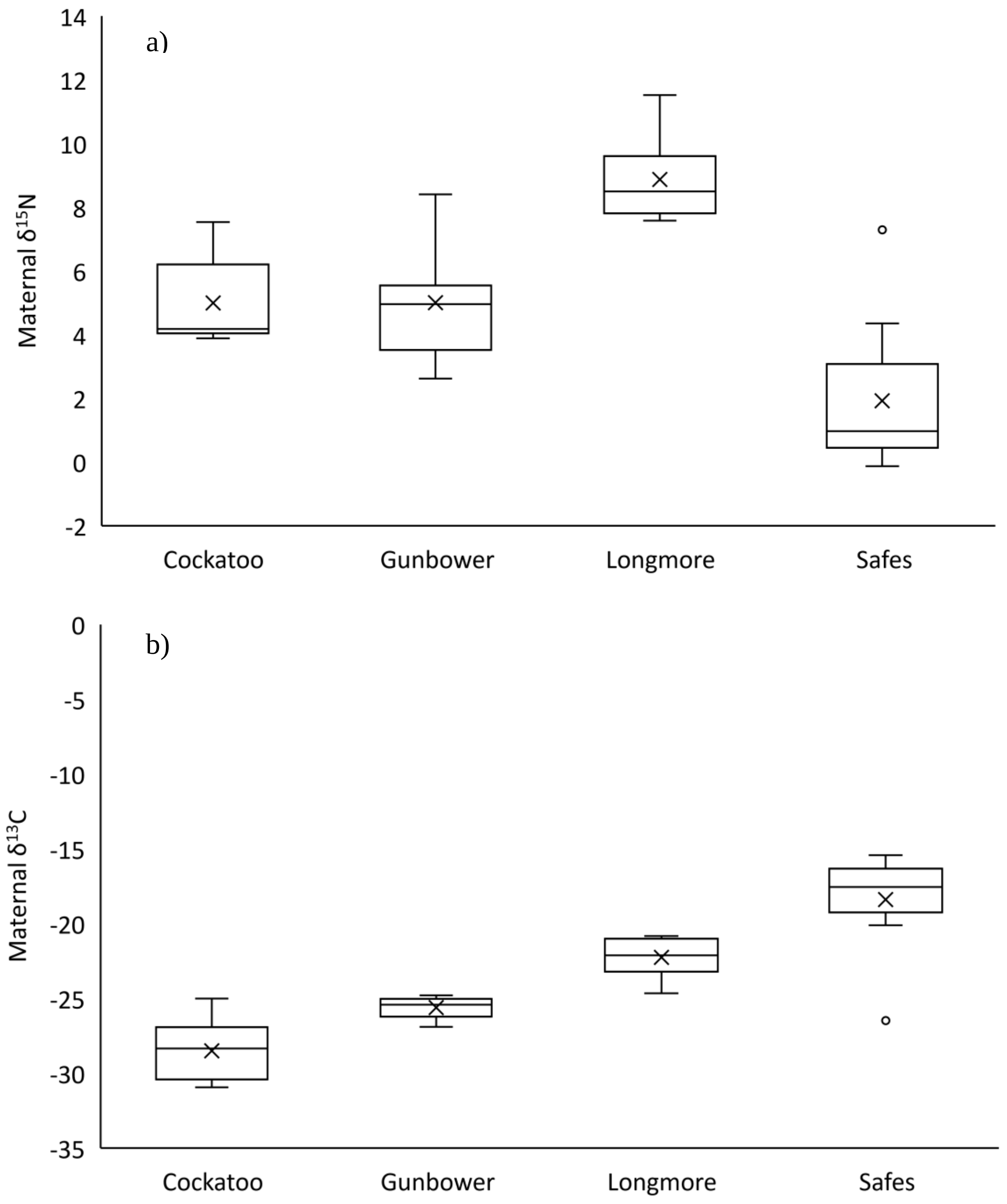

Figure S1. Differences in a) maternal $\delta^{15}N$ and b) maternal $\delta^{13}C$ across sites.

Table S6. Size and composition data of eggs sampled from gravid *E. macquarii* from Cockatoo Lagoon.

Maternal ID	Maternal body size (SCL; mm)	Maternal body mass after ovipositing (kg)	Clutch size	Total clutch mass (g)	Laying order	Egg mass (g)	Egg width (mm)	Egg length (mm)	Egg water content (g)	Egg lipid content (g)	Egg total energy (kJ)	Egg bulk protein mass (g)
CL1	286	2.18	24	195.64	First	8.29	20.12	35.04	6.6053	0.1539	28.1854	0.6330
CL1	286	2.18	24	195.64	Last	8.61	20.64	34.6	6.9111	0.1435	39.4998	0.7472
CL2	260	1.88	20	174.78	First	8.65	21.17	33.09	6.9184	0.1519	42.0537	0.6718
CL2	260	1.88	20	174.78	Last	8.8	20.57	35.41	6.9782	0.1581	44.1553	0.8597
CL3	258	1.885	11	119.31	First	10.72	21.88	38.19	8.3255	0.4894	54.3569	1.1028
CL3	258	1.885	11	119.31	Last	10.86	21.29	37.73	8.3017	0.3327	61.9905	0.9623
CL4	268	2.175	15	145.54	First	9.11	28.86	34.94	7.0399	0.2976	58.3262	0.9515
CL4	268	2.175	15	145.54	Last	10.93	21.18	39.49	8.6598	0.3042	54.0714	0.9784
CL5	274	2.08	29	250.11	First	8.96	20.19	35.12	7.0374	0.1515	37.1184	0.8340
CL5	274	2.08	29	250.11	Last	7.59	20.07	32.75	6.2478	0.1540	28.8433	0.5610
CL6	273	2.26	17	183.77	First	10.97	21.45	38.45	8.4487	0.5029	58.1784	0.9453
CL6	273	2.26	17	183.77	Last	11.65	21.63	39.12	8.8123	0.4763	66.7697	1.3972
CL7	266	2.085	16	170.21	First	10.69	21.97	37	8.4732	0.2764	52.0719	1.1086
CL7	266	2.085	16	170.21	Last	10.62	21.66	37.56	8.3682	0.2935	53.7673	0.9609

Table S7. Size and composition data of eggs sampled from gravid *E. macquarii* from Gunbower Creek.

Maternal ID	Maternal body size (SCL; mm)	Maternal body mass after ovipositing (kg)	Clutch size	Total clutch mass (g)	Laying order	Egg mass (g)	Egg width (mm)	Egg length (mm)	Egg water content (g)	Egg lipid content (g)	Egg total energy (kJ)	Egg total bulk protein mass (g)
GC1	276	2.1	20	203.56	First	9.84	21.11	37.11	7.8	0.1987	42.7569	0.6295
GC1	276	2.1	20	203.56	Last	10.51	21.71	37.3	8.7357	0.1614	41.6099	0.7408
GC2	271	2.215	23	217.88	First	9.1	20.53	35.09	7.1388	0.1562	50.1551	0.9475
GC2	271	2.215	23	217.88	Last	8.86	20.11	34.68	7.0715	0.2932	41.6738	0.7664
GC3	257	2.005	15	156.34	First	10.68	21.56	37.64	7.8566	0.5791	63.5095	1.1119
GC3	257	2.005	15	156.34	Last	9.77	20.52	38.45	7.278	0.3919	63.0992	1.3145
GC4	251	1.59	14	137.90	First	9.21	21.68	33.07	6.9246	0.5087	53.3101	1.1455
GC4	251	1.59	14	137.90	Last	9.87	22.01	34.68	7.6471	0.2279	51.2903	1.0715
GC5	276	2.3015	21	250.84	First	10.66	22.25	36.08	8.1198	0.1928	60.5093	1.1401
GC5	276	2.3015	21	250.84	Last	12.99	23.14	39.15	10.0403	0.3008	65.9349	1.0309
GC6	257	2.02	18	211.16	First	11.63	22.25	37.3	9.0292	0.4203	56.9303	1.2054
GC6	257	2.02	18	211.16	Last	11.23	22.12	37.01	9.0092	0.3453	52.9320	1.0683
GC7	251	1.63	17	149.84	First	8.7	20.78	34.53	7.009	0.3541	37.6524	0.7138
GC7	251	1.63	17	149.84	Last	8.88	20.59	35.07	7.1773	0.2528	40.3452	0.7955

Table S8. Size and composition data of eggs sampled from gravid *E. macquarii* from Longmore Lagoon.

Maternal ID	Maternal body size (SCL; mm)	Maternal body mass after ovipositing (kg)	Clutch size	Total clutch mass (g)	Laying order	Egg mass (g)	Egg width (mm)	Egg length (mm)	Egg water content (g)	Egg lipid content (g)	Egg total energy (kJ)	Egg total bulk protein mass (g)
LM10	310	3.175	20	221.61	First	11.04	22.17	37.51	9.0352	0.2328	43.5914	1.0466
LM10	310	3.175	20	221.61	Last	11.02	22.08	38.01	8.8234	0.2210	51.6146	1.0513
LM11	294	2.775	21	200.97	First	9.66	28.84	36.88	7.8883	0.3964	41.0705	0.6307
LM11	294	2.775	21	200.97	Last	9.19	20.87	35.5	7.2682	0.2627	44.9943	0.6637
LM13	278	2.25	18	160.39	First	7.75	20.08	32.47	6.1439	0.2751	39.4054	0.7814
LM13	278	2.25	18	160.39	Last	9.55	21.3	34.58	7.6461	0.3162	46.0732	0.9237
LM14	264	2.1	16	165.63	First	9.9	20.95	37.21	7.2593	0.3699	58.3363	1.1629
LM14	264	2.1	16	165.63	Last	10.78	21.01	39.45	8.1648	0.2401	57.4119	0.9881
LM2	284	2.59	18	200.27	First	11.32	22.9	35.55	8.7333	0.3761	61.3116	1.1904
LM2	284	2.59	18	200.27	Last	10.78	22.24	36.17	8.3025	0.2203	58.0385	1.1662
LM4	298	2.94	18	205.82	First	10.93	22.18	37.56	8.7185	0.3676	58.1339	1.0498
LM4	298	2.94	18	205.82	Last	11.82	22.7	38.53	9.1281	0.4730	63.7952	0.8863
LM5	270	1.92	16	180.00	First	10.64	21.47	37.89	8.4132	0.3352	56.4461	0.9797
LM5	270	1.92	16	180.00	Last	12.05	22.63	39.47	9.6393	0.1962	61.3616	1.2126
LM8	270	2.385	19	225.38	First	11.96	22.73	39.51	9.3587	0.2616	59.6267	1.0670
LM8	270	2.385	19	225.38	Last	12.24	23.21	37.99	9.6447	0.4402	60.5797	1.1132

Table S9. Size and composition data of eggs sampled from gravid *E. macquarii* from Safes Lagoon

Maternal ID	Maternal body size (SCL; mm)	Maternal body mass after ovipositing (kg)	Clutch size	Total clutch mass (g)	Laying order	Egg mass (g)	Egg width (mm)	Egg length (mm)	Egg water content (g)	Egg lipid content (g)	Egg total energy (kJ)	Egg total bulk protein mass (g)
SL1	265	2.05	19	196.81	First	10.26	21.48	36.71	8.0586	0.5598	50.3958	0.7253
SL1	265	2.05	19	196.81	Last	10.58	21.44	37.77	8.4671	0.3209	46.3210	0.7749
SL2	249	1.685	14	132.57	First	9.38	20.71	36.86	7.8076	0.2354	34.8914	0.6440
SL2	249	1.685	14	132.57	Last	9.1	20.5	36.19	6.9452	0.1660	51.3676	0.9327
SL3	256	1.88	18	167.08	First	8.33	19.53	35.69	6.3193	0.3209	48.2019	0.9367
SL3	256	1.88	18	167.08	Last	10.16	21.02	37.75	8.2979	0.4197	44.1073	0.8708
SL4	285	2.625	22	234.28	First	10.41	22.73	33.94	7.6646	0.4137	65.7118	1.2163
SL4	285	2.625	22	234.28	Last	10.89	22.98	35	8.3893	0.5293	59.3818	1.1512
SL5	265	2.07	21	217.77	First	9.82	20.74	37.41	7.4348	0.2391	54.2249	0.9978
SL5	265	2.07	21	217.77	Last	10.56	20.44	39.78	7.9863	0.3478	58.7447	1.2578
SL6	262	2.02	22	198.73	First	9.41	20.86	35.88	7.4801	0.3200	44.2901	0.6088
SL6	262	2.02	22	198.73	Last	8.88	19.91	36.74	6.8799	0.3136	47.2496	0.7040
SL7	268	2.355	22	216.66	First	9.79	21.51	35.14	7.7352	0.3180	50.1829	1.1616
SL7	268	2.355	22	216.66	Last	9.57	21.45	34.9	7.3338	0.3655	52.1535	1.1142
SL8	250	1.685	20	148.66	First	7.6	19.7	32.32	5.9552	0.1248	38.6870	0.6883
SL8	250	1.685	20	148.66	Last	7.42	19.55	32.54	5.6181	0.2859	42.3152	0.6285
SL9	243	1.43	12	107.46	First	8.69	21.09	33.13	7.1397	0.3481	38.4326	0.6921
SL9	243	1.43	12	107.46	Last	8.66	21.15	33.46	7.0466	0.5038	31.4871	0.7479

Table S10. Linear equations for the relationship between total clutch mass and maternal body size, for each site.

Site	Estimate	y-intercept
Cockatoo	4.199 ± 1.832	- 7.967 ± 4.444
Gunbower	4.466 ± 1.602	– 8.534 ± 3.874
Longmore	1.458 ± 1.272	– 1.289 ± 3.088
Safes	5.110 ± 0.980	– 10.098 ± 2.366

Table S11. Analysis of covariance comparing the relationships between water content of the eggs, site, laying order, log-transformed egg dry mass, and full factorial interactions.

Effect	Num *df*	Den *df*	*F*	*P*
Site	3	19	0.66	0.585
Laying order	1	19	0.02	0.901
Site x Laying order	3	19	0.52	0.676
Egg dry mass*	1	19	22.10	0.001
Egg dry mass x Site	3	19	1.01	0.410
Egg dry mass x Laying order	1	19	0.22	0.648
Egg dry mass x Site x Laying order	3	19	0.45	0.722

Table S12. Analysis of covariance comparing the relationships between protein content of the eggs, site, laying order, log-transformed egg dry mass, and full factorial interactions.

Effect	Num *df*	Den *df*	*F*	*P*
Site	3	19	0.62	0.612
Laying order	1	19	0.43	0.522
Site x Laying order	3	19	0.50	0.686
Egg dry mass*	1	19	58.03	<0.001
Egg dry mass x Site	3	19	0.56	0.646
Egg dry mass x Laying order	1	19	0.32	0.575
Egg dry mass x Site x Laying order	3	19	0.52	0.673

Table S13. Analysis of covariance comparing the relationships between lipid content of the eggs, site, laying order, log-transformed egg dry mass, and full factorial interactions.

Effect	Num *df*	Den *df*	*F*	*P*
Site	3	18	3.16	0.050
Laying order	1	18	0.46	0.508
Site x Laying order	3	18	0.60	0.623
Egg dry mass*	1	18	18.90	0.001
Egg dry mass x Site	3	18	2.81	0.069
Egg dry mass x Laying order	1	18	0.82	0.377
Egg dry mass x Site x Laying order	3	18	0.61	0.618

Table S14. Analysis of covariance comparing the relationships between energy content of the eggs, site, laying order, log-transformed egg dry mass, and full factorial interactions.

Effect	Num *df*	Den *df*	*F*	*P*
Site	3	19	1.94	0.157
Laying order	1	19	0.31	0.586
Site x Laying order	3	19	1.88	0.168
Egg dry mass	1	19	313.43	<0.001
Egg dry mass x Site	3	19	1.80	0.182
Egg dry mass x Laying order	1	19	0.18	0.679
Egg dry mass x Site x Laying order	3	19	1.40	0.273

Table S15. Analysis of covariance comparing the relationships between water content of the eggs, site, laying order, and log-transformed egg dry mass. Asterisks indicate significant effects.

Effect	Num *df*	Den *df*	*F*	*P*
Site	3	29	1.89	0.154
Order	1	29	2.08	0.160
Egg dry mass *	1	29	24.15	<0.001

Table S16. Analysis of covariance comparing the relationships between the protein content of the eggs, site, laying order, and log-transformed egg dry mass. Asterisks indicate significant effects.

Effect	Num *df*	Den *df*	*F*	*P*
Site	3	29	0.14	0.937
Order	1	29	0.09	0.768
Egg dry mass *	1	29	72.91	<0.001

Table S17. Analysis of covariance comparing the relationships between the lipid content of the eggs, site, laying order, and log-transformed egg dry mass. Asterisks indicate significant effects.

Effect	Num *df*	Den *df*	*F*	*P*
Site	3	28	1.00	0.408
Order	1	28	1.02	0.321
Egg dry mass *	1	28	16.17	0.001

Table S18. Analysis of covariance comparing the relationships between the energy content of the eggs, site, laying order, and log-transformed egg dry mass. Asterisks indicate significant effects.

Effect	Num *df*	Den *df*	*F*	*P*
Site	3	29	0.34	0.798
Order	1	29	0.25	0.620
Egg dry mass *	1	29	345.45	<0.001

Table S19. Generalised linear mixed model testing the effects of site, temperature and the interaction of site and temperature, on hatching success rates.

Effect	Num *df*	Den *df*	*F*	*P*
Site	3	28	2.12	0.121
Temp	1	28	0.04	0.840
Site x Temp	3	28	1.19	0.332
